# Supplementary material for: Podocytopathy Associated with IgA Nephropathy in Pregnancy: A Challenging Association
Source: J Clin Med. 2023 Feb 27;12(5):1888. doi: 10.3390/jcm12051888 (PMC10004185; doi:10.3390/jcm12051888)
Supplement: Supplementary file 1 [file jcm-12-01888-s001.zip › jcm-2195906-supplementary.pdf]

Supplemental Figure S1: (HE, 400x)

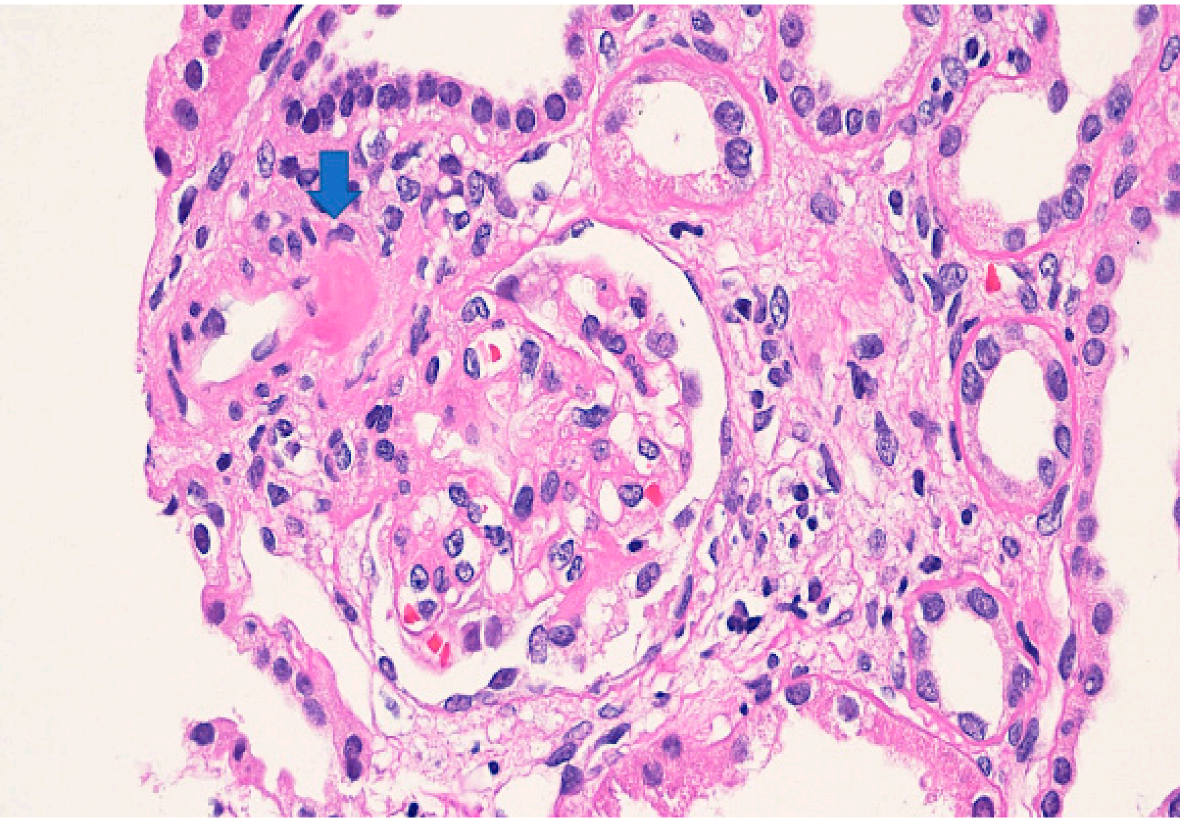

Figure S1. Preglomerular and interstitial arteriolar vessels have moderate circumferential wall thickening, with hypertrophy of the media (supplemental Figure S1, blue arrow).
